# Supplementary material for: Enhancing Image-Guided Radiation Therapy for Pancreatic Cancer: Utilizing Aligned Peak Response Beamforming in Flexible Array Transducers
Source: Cancers (Basel). 2024 Mar 22;16(7):1244. doi: 10.3390/cancers16071244 (PMC11011135; doi:10.3390/cancers16071244)
Supplement: Supplementary file 1 [file cancers-16-01244-s001.zip › cancers-2852676-supplementary.pdf]

Supplementary results

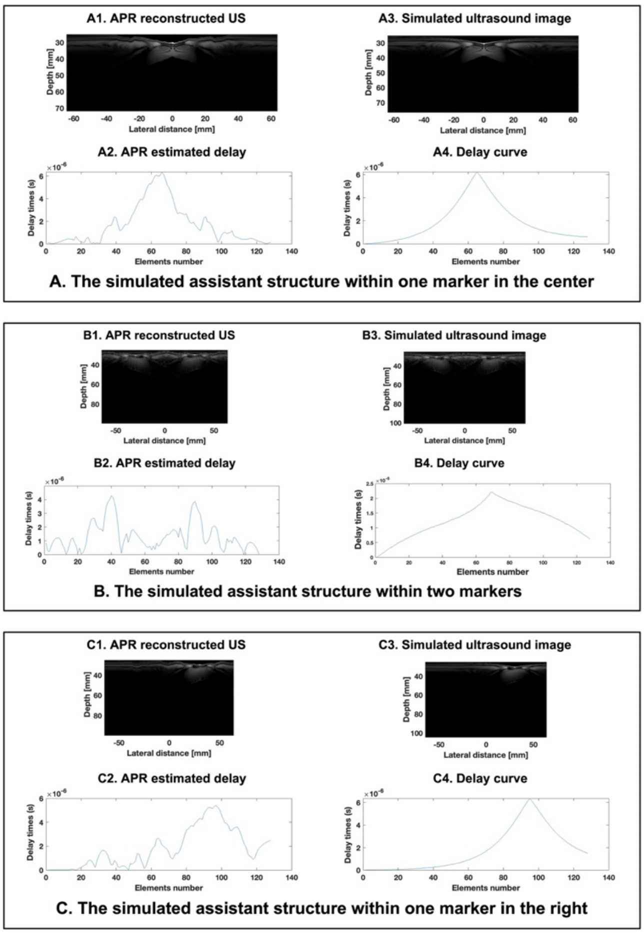

Figure S1. The APR results based on the Field-II simulated raw data from different simulated point target phantoms. A1 and A2 show the simulated ultrasound images of one point target in the center of the phantom after APR delays and the corresponding delay curve of the point target phantom by APR estimation. A3 and A4 show the Ground Truth of the simulated ultrasound image of this phantom and corresponding delay curve. B1 and B2 show the simulated ultrasound images of two symmetric point targets after APR delays and the corresponding APR estimated delay curve of the two symmetric point targets. B3 and B4 show the Ground Truth of the simulated ultrasound image and the delay curve for this phantom. C1 and C2 show the simulated ultrasound images of one point target in the right side of the phantom after APR delays and the corresponding APR estimated delay curve of the one-point target on the right side of the phantom. C3 and C4 show the Ground Truth of the simulated ultrasound image and the delay curve for this phantom. The radius of curvature of the simulated flexible probe is 700 mm. All simulated ultrasound images are showed in [-60 0] dB.

Figure S1 shows the APR results based on the Field-II simulated raw data from different simulated point target phantoms. Figure 1. A1 and A2 show the simulated ultrasound images of one point target in the center of the phantom after APR delays and the corresponding APR estimated delay curve of the point target phantom. The x-axis and y-axis represent the element's number and the amount of delay times, respectively. Figure S1. A3 and A4 display the ground truth of the simulated ultrasound image of this phantom, which is reconstructed by using conventional DAS method based on the known flexible transducer shape (a linear one) and the corresponding delay curve. Similarly, figure S1. B1 and B2 show the simulated ultrasound images of two symmetric point targets after APR delays and the corresponding APR estimated delay curve of the two symmetric point targets. Figure S1. B3 and B4 display the ground truth of the simulated ultrasound image of this phantom, which is reconstructed by using conventional DAS method based on the known flexible transducer shape (a linear one) and the corresponding delay curve. Similarly, figure S1. C1 and C2 show the simulated ultrasound images of one point target in the right side of the phantom after the APR

delays and the corresponding APR estimated delay curve of the one-point target on the right side of the phantom. Figure S1. C3 and C4 display the ground truth of the simulated ultrasound image of this phantom, which is reconstructed by using conventional DAS method based on the known flexible transducer shape (a linear one) and the corresponding delay curve. By comparing the simulated ultrasound image results and delay curve after APR delays with their corresponding ground truth images and delay curve, it can be observed that the APR method performed optimally when there was only one point target in the center of the simulated phantom. All simulated ultrasound images were shown in the [-60 0] dB dynamic range. The radius of curvature of the simulated flexible probe is 700 mm. The results indicate the estimation of APR is more accurate with the simulated point phantom included one center point marker. Compare to the results based on simulated point phantom with two markers or one right point marker, the APR estimated delay curves estimated based on one center point marker phantom are closer to the ground truth delay curve. The total error delays of one point target, two symmetric point targets, and one-point target on the right side of the phantom are $5.2691\times10^{(-5)}$ s , $1.2096\times10^{(-4)}$ s, and $6.3110\times10^{(-5)}$ s, respectively. The corresponding mean error is $4.1160\times10^{(-7)}$ s , $9.4500\times10^{(-7)}$ s , and $4.9310\times10^{(-7)}$ s , respectively. The results of scatter phantom simulations are showed in the supplementary files.
